# Gleanings from Our Exchanges

**Published:** 1872-10-01

**Authors:** 


### Gleanings from Our Exchanges.

### A CASE OF HYDROCELE, WITH CARTILAGINOUS THICKENING OF THE TUNICA VAGINALIS.

BY S. E. MUNFORD, PRINCETON, IND.

Mr. T., age 54, received a kick from a horse fifteen years ago, there resulting a slight contusion of the scrotum and left testicle. Some swelling followed, which continued for two years and disappeared. Soon, however, the scrotum began enlarging again, and in a few years there was a tumor of such size and weight as to prove a source of great inconvenience. About this time he consulted a surgeon, who told him there was malignant disease of the testicle, and that nothing save castration would relieve him. The proportions of the remedy were greater than he felt willing to submit to, and as a consequence, did nothing for a few years more. The tumor in the meanwhile had been growing, and when I saw him in February, 1871, it was evident from his failing health that definite action in the case could be no longer delayed.

On the 11th of February, Dr. West, his family physician, and myself, visited him, for the purpose of determining, if possible, the nature of the trouble, and deciding upon a plan of treatment. Mr. T. is a man of large frame, being six feet two inches high, and weighing, when in health, two hundred and twenty-five pounds.

We found the tumor twelve and one-half inches in length, and at the largest part, which was a little above the middle, twenty-four inches in circumference. The skin over a small portion of the anterior and upper surface was smooth and elastic, elsewhere it was slightly wrinkled. The general surface of the swelling was irregular, being in some places distinctly nodular, and to the touch gave a sensation of solidity, save over the portion having the tense, smooth skin, which gave the slightest sensation of fluctuation. A careful examination in a darkened room showed no translucency.' The penis was entirely submerged, its investing skin being drawn into the scrotum.

Dr. Patten, of Princeton, saw the case with us the following day. An exploring trocar was introduced, and this revealing the presence of fluid, a large flat trocar and canula were pushed into the tumor at its middle and anterior surface. Two quarts and eight ounces of a dark brown fluid were drawn off. In passing the trocar, it was discovered that the vaginal tunic was cartilaginous and much thickened. So great was this thickening, the walls could not be sufficiently compressed to completely evacuate the fluid, and we were satisfied a considerable quantity remained after our manipulations.

The second day from the tapping, the sack began filling again, and on the fourth was apparently as large as ever. Introduced the trocar and canula again, fully expecting to find as large a quantity of fluid as on the first occasion. It measured, however, but thirty ounces, was tolerably clear, and on standing a few moments was filled with flakes of lymph. This we accepted as evidence of an acute inflammation of the serous lining, and gave him for a few days such antiphlogistic treatment as we thought necessary to modify the inflammatory action. After a certain reduction in size had taken place, and all the febrile symptoms had subsided, the tumor became stationary.

Thinking it depended upon fluid, the trocar was again introduced, and notwithstanding the canula was pushed to its shoulder, not one drop of fluid came away. It was apparent from this that the fluid had been wholly absorbed, and that only the dense fașcia and thickened sack were to be disposed of by a process of absorption. The measurements at this time were : length, nine inches, circumference, seventeen and one-half inches. Emollient applications were made, and the enlarged veins of the scrotum opened every morning for several days in succession. As soon as it was found the tumor was softening, it was bound up by means of strips of heavy moleskin plaster, so as to compress it equally at every point. Happily the absorbents began their work again, and in six months from the first tapping the cure was in every way complete.

A few points in the case suggested this report :

1st. The quantity of fluid as being much beyond the average for this climate, and the size of the tumor as being altogether out of proportion to its contents.

2d. The cure as having followed only palliative treatment, contrary to what might have been expected in a case of such long standing and having such marked structural changes.

3d. The difficulties involved in diagnosis. There was a departure in several essential ways from ordinary hydrocele. Instead of a tumor that was smooth, elastic, oval, or pyriform, fluctuating and transparent, the skin, with the exception of a small surface, was rugous, the tumor to the touch had a sensation of solidity, was irregular or nodular, fluctuating indistinctly at one part only, and perfectly opaque.

In all cases of long standing in which there is thickening of the sack, this difficulty will be encountered. The point is to distinguish between hydrocele and malignant disease of the testicle, and nothing save an exploratory puncture by a delicate trocar or grooved needle, will decide as between them. Even if disease of the testicle exists, the wound of so delicate an instrument can do no harm. A surgeon, well known in a large section of country as having distinguished ability, pronounced this case encephaloid of the testicle. Curling, in his work on Diseases of the Testes, relates a case occurring in his practice that he was unable to decide, until his patient having died of disease of the chest, a *post-mortem* examination revealed an old hydrocele with cartilaginous thickening of the tunic. He also relates a case, having undergone the same textural changes, occurring with Dupuytren. It had all the features of scirrhous disease, was unaccompanied with any sign indicative of hydrocele, and this distinguished surgeon was only able to decide after having made an exploring puncture.—*Trans. Ind. State Med. Soc.*

TINCTURE OF CALABAR BEAN.—By R. ROTHER.—Eserine,the alkaloid,is the active principle of calabar bean. The leguminous seed contains it closely enveloped within its substance, the bulk of which is starch and legumin or vegetable casein. The alkaloid occurs in nature partially combined as a salt. Alcohol, strong or dilute, extracts the virtue of the bean, but water alone effects this less completely. When treated with strong alcohol the substance must be in a very fine powder, and requires a preliminary digestion with the menstruum before proceeding with the percolation, otherwise the exhaustion will not be perfectly attained. The original solid material is impervious to strong alcohol, and since the solvent effect of this depends upon surface action mainly, hence a coarse powder is wholly inapplicable for the purpose of extraction with this liquid. The pulverization of calabar bean is rather difficult in a small way, on account of its hardness and peculiar structure, and, moreover, great care must be exercised in the operation to avoid dusting, by reason of the poisonous character of the drug; consequently, a process which admits the application of a coarse powder together with a weaker alcohol, would be much more desirable than the method in present use.

The employment of acetic acid as part of the menstruum, results in the conversion of the alkaloid into soluble acetate; but a weak alcohol directly applied to a coarse powder does not appear to act favorably; the soluble legumin and the unchanged starch interfere with the process.

Water swells the material, dissolving the legumin, and if the mixture is allowed to stand in a warm place for a few days it will become putrid. If, however, a small quantity of acetic acid be first added, the legumin will not dissolve, but shortly the vinous fermentation will begin, resulting in the copious evolution of carbonic acid, the disappearance of the starch and total disintegration of the original structure. By this change the alkaloid is exposed and easily and completely extracted by the acetic acid. After a few days, and rather before the end of the vinous fermentation, an equal volume of strong alcohol is added to the mixture and the maceration continued several days. It now becomes necessary to separate the liquid from the insoluble residue and complete the exhaustion. The process is therefore finished by percolation. For this purpose it becomes necessary, which indeed is a decided advantage in all percolations, to produce a solid substratum of coarse powder. Such is effected in this case by pouring part of the mixture upon a muslin strainer, separating the liquid by pressure and forming a solid layer several inches high in the bottom of the percolator with the residual remnant on the strainer. Upon this foundation the remaining mixture is poured, together with the strained liquid, and then followed by a mixture composed of equal measures of strong alcohol and water until the required volume of percolate has passed. The preparation has a deep amber tint.

The tincture should conform in the proportion of the solid material with most other tinctures, and therefore contain two troy ounces of the bean to the pint of finished product.

The process is as follows:

℞ Calabar Bean, No. 50 powder, 16 ℥ tr.

Acetic Acid, ½ fl ℥

Strong Alcohol,

Water, aa sufficient.

Mix the acid with three pints of water; pour this upon the powder and set the mixture aside for a few days in a warm place. Then add to it three pints of strong alcohol and macerate it for a few days longer. After this pour part of the mixture upon a muslin strainer; press the liquid out, and pack the residue of the strainer firmly into a cylindrical glass percolator having a broad base; pour on to this foundation the rest of the mixture together with the strained liquid, and then a mixture of equal parts of strong alcohol and water, until eight pints of percolate has passed.—*The Pharmacist.*

A Fatal Case of Hysteria.—It is but rarely that an opportunity is offered for an examination of the *post-mortem* appearances of the organs of persons who have suffered from this affection, and we therefore transcribe the notes of a case published in the *Brit. Med. Jour.* of July 6th. About three o'clock p. m. of October 11th, 1870, Dr. R. W. Foss, of Stockton-on-Tees, was called to see a young lady, aged nineteen, who had been having convulsions all day. At the time of the visit she was perfectly conscious, throwing her arms and legs about, laughing and crying alternately, in fact, having hysterical convulsions. She complained of pain in the right side of her head, also of the *globus hystericus* rising up from her abdomen to the right side of her chest, where it stopped. Her chest gave negative physical signs; pulse about 80, and small. She stated that she had been having leucorrhœa with much offensive discharge for the last fourteen months, and within that time had had these hysterical attacks. All of this time she had been under medical care. Her father stated that all the physicians who had seen her had said that she was simply suffering from hysteria with leucorrhœa, and that in time she would be well again. The same opinion was expressed by Dr. Foss, who believed that it was a case for moral more than for medical treatment. Within three hours from Dr. Foss's visit she was dead, and at the desire of her father a *post-mortem* examination was held, with the following results:

The lungs were normal, except some hypostatic congestion at their bases, and the presence of some frothy mucus in the bronchial tubes. The heart was pale and rather flabby, the apex of the right ventricle containing a small clot; the other cavities were empty. The liver seemed rather larger than normal, but appeared healthy on section. No disease discovered in the kidneys or supra-renal capsules. The contents of the cranium appeared perfectly natural. The base of the uterus was congested, and its walls were thicker than usual. The mucous membrane lining its interior was covered with a thick glassy mucus, like white of egg, which was also exuding from the os. The left ovary was about the usual size, and, on section, presented a honeycombed appearance, consisting of numerous little cavities filled with gelatinous matter, constituting what is called "colloid tumor of the ovary." The right ovary was the size of a large walnut, and, on section, was seen to consist of one large parent cyst containing many smaller ones springing from its internal surface. There was nothing here to account for so sudden a death, and the only hypothesis offered was, considering the condition of the heart, that death was caused by syncope.

A nearly similar case is reported to have occurred in 1868 at the Royal Infirmary in Edinburgh, in the person of a strong, well-developed woman, between 20 and 30 years of age, in which, on *post-mortem* examination, none of the organs were found to be diseased.—*Med. Record.*

Diphtheritic Albuminuria.—R. Browning, L. R. C. P. L., in the *British Medical Journal*, says: From what I have lately witnessed while watching two local epidemics of diphtheria, I am disposed to consider that albuminuria is present in nearly all cases. That its appearance is usually about the end of the first week after the diphtheritic membrane is developed, though sometimes earlier more rarely later. Co-existently with its appearance, there is a notable diminution of the quantity of urine, and an increased excretion of urea; whilst lithates generally, tube casts, both granular and waxy frequently, blood corpuscles not seldom, and pus globules occasionally, are found on examination of what is secreted. The urinary specific gravity mostly averages 1016, and the temperature of the body is, as a rule, 100.4 to 102 degrees.

The gravity of the prognosis increases in an equal ratio with the quantity of albumen existing in the urine, independently of the amount of throat affection or kidney disorganization, and an early or late discovery of albumen is of serious import. The local mischief attacking the pharynx, larynx, or other structures, and paralysis subsequently occurring, are entirely the result and symptomatic of a morbid poison affecting the general system, just as the sore throat of syphilis is the sequence of a blood disease previously contracted. Albuminuria, in any quantity, is due to obstruction of circulation through the kidneys, caused by congestion of the malpighian tufts, this congestion being produced by paralysis of the nerves supplied to them; but a mere trace only of albumen arises either from pus or else blood which has casually entered the volume of urine. The indication of treatment is to remove this obstruction by overcoming the paralysis; and this is best accomplished by local Faradization. Seven cases are reported in detail, two of which terminated fatally. In these two no Faradization was employed. The other five, which were all of a very serious nature, recovered after Faradization was resorted to. All were marked by unmistakable evidence of blood poisoning and albuminuria, with more or less suppression of urine. The treatment of all was conducted on the same principles, plus or minus the induction coil; the object aimed at being at first, during the premonitory symptoms, to regulate the secretions, and then to support the strength of the system in every possible way. My sheet anchor was the tincture of perchloride of iron, sometimes combined with glycerine, sometimes with chlorate of potash, and sometimes given *per se.* Stimulants and nourishment in every variety were supplied with no sparing hand. The customary topical medication was of course attended to. In some instances, the ordinary conductors fitted to most galvanized batteries; in others "Etna's" were employed. Faradism was thus employed over the lumbar regions along the lower part of the spine, and as nearly as possible in the direction of the ureters.—*Detroit Review.*

Cases of Double Ovariotomy.—Prof. T. Gaillard Thomas, M. D., New York (*American Practitioner*, July, 1872), reports three successful cases of double ovariotomy, which were performed in the Strangers' Hospital of this city, after an experience of twenty-seven operations of ovariotomy—fourteen of which have already appeared in print. The remaining thirteen are being prepared for publication.

Incontinence of Urine in Children.—Holmes Coote recommends for this intractable affection the administration of creasote in one-minim doses, three times daily, combined with asafœtida and rhubarb pill, of each two grains.

Tracheotomy.—Dr. P. Landi reports a successful case of tracheotomy for the extraction of a first molar tooth, lodged for twenty-four hours in the left bronchus of a child seven years of age; the tooth having escaped from the forceps at the moment of extraction.—*Med. Record.*

Veratrum Viride as a Hemostatic.—Dr. J. W. Collins, in the *American Practitioner*, says of veratrum viride, that he esteems it the promptest as well as the most reliable of all our means for controlling both active and passive hemorrhage. A case of hemoptysis which had resisted all the usual remedies, yielded almost at once to five drops of Norwood's tincture given every hour for six hours. A case of aneurism of the subclavian artery, involving all the second portion, and probably the first also, was so much benefited by this remedy that at the end of ten days the patient considered himself cured, and imprudently quit visiting the doctor. At the end of two weeks he returned with most of the symptoms as well marked as at first. He was put upon the same treatment, five drops, increasing one drop every three hours, until the full sedative effect was produced. This was continued for fifteen days, the last six days merely to make sure of a cure, for the tumor, with all the attending symptoms, had disappeared, the radial pulse had become normal, and the natural temperature of the extremity restored. At the end of twelve months when seen again, he was perfectly well. In epistaxis veratrum was uniformly successful, so also in menorrhagia. It arrested hemorrhage in two cases of carcinoma; saved the life of a patient where secondary hemorrhage took place after amputation of the cervix uteri; arrested secondary hemorrhage following amputation of the thigh.—*Detroit Review.*

Epithelioma Cured by Creasote.—Dr. Forne reports, in the *Montpellier Medical*, a case of epithelioma of the upper lip of about four centimeters in diameter, cured by the topical application of creasote. The treatment was commenced by dipping a brush in pure creasote, and having removed any that might drop, the whole surface of the ulcer was lightly but firmly touched with the brush, a certain degree of pressure being used, and the brush retained on the same point a few seconds, so as to insure a thorough application. A piece of lint moistened in a gummy solution of creasote was then laid upon the ulcer. The application caused but slight pain. Three days subsequently it was found that the ulcer had not increased. The application was repeated in the same manner, and five times afterwards, at about the same interval. Cicatrization then commenced, and proceeded rapidly, being favored by a dressing of thin paper moistened with a solution of creasote. Entire recovery took place about six weeks after the treatment was commenced.—*Med. and Surgical Reporter.*

Treatment of Ulcers of the Leg.—Mr. W. E. C. Nourse, of Brighton, reports in the *British Medical Journal*, that he has treated nearly five hundred cases of ulcerated legs during the last thirteen years, and that the results of his treatment have been neither unsatisfactory nor disheartening, having met with only ten or fifteen failures out of the whole number. The plan of treatment has been the application of uniform pressure, with strappings, bandages, etc.; the sparing use of stimulating ointments or lotions to the ulcers, and of internal medicines, and the unsparing use of pains and trouble to do the work properly; the non-disturbance of the healing process by frequent dressings, the wounds being uncovered only once, or at most twice a week; the recognition of a low vitality as a common cause in most of these cases, involving more or less destruction of tissues, and the consequent avoidance of mercury, strong or frequent cathartics, and all forms of depressing medicines, and the allowing to the patient his ordinary diet and mode of living, cutting off only whatever seemed excessive or positively injurious, and the removal from the limb of all ligatures or anything likely to obstruct the circulation, also of irritant or injurious applications.—*Medical Times.*

A Diagnostic Sign of Extra Capsular Fracture of the Neck of the Femur.—In a recent monograph upon the subject, M. Jankergusitel arrives at the following conclusions:

1. Increase in the size of the great trochanter is an accurate and constant sign of extra capsular fracture of the neck of the femur.
2. The presence of this sign once ascertained enables the surgeon to dispense with all those manipulations, often dangerous, always painful, which are necessary to determine crepitation or abnormal mobility.
3. The study of the sign alluded to permits accuracy of diagnosis, without rendering the prognosis more grave, or without compromising a case.—*Boston Medical and Surgical Journal.*

Sal Ammoniac in Dropsy—Spencer Thomson, M.D., Ashton, Torquay, finds the hydrochlorate of ammonia one of the most efficient of remedies in cases of portal dropsy, prescribed in scruple or half-drachm doses, given tolerably diluted, every six or eight hours. The recent accounts of the success attending the employment of this remedy in hepatic affections in India, he hopes will lead to its more extensive use in England.—*The Clinic.*

A Mode of Operating for Radical Cure of Varicocele. — Dr. H. B. Davison, San Francisco (*Pacific Med. and Surg. Journal*), has adopted a new mode of operating for the radical cure of varicocele, for which he claims three great advantages over any other means:

First, by perforating only one wall of the scrotum, less pain, less inflammation, and less risk of adhesion of the wounded sac and spermatic cord.

Second, by placing the patient in a recumbent posture when the operation is being performed, so that no blood may be enclosed in that portion of the vein cut off from the circulation, the resultant inflammation will be much less and the testicle will not swell so much, and absorption will be accomplished in much less time.

Third, by removing the ligature before it cuts through the vein, the risk of phlebitis is lessened, and the patient is enabled to resume his ordinary duties much sooner.

Those who have been operated on have no return of the disease, and it would require a very close examination of the parts to discover that any operation had been performed. In one case the patient had been wearing a suspensory bandage for over twenty years, and the left testicle was much atrophied. It is now about sixteen months since the operation, and the testicle has regained its normal size, and the patient has a corresponding increase of sexual power.—*Ibid.*

The Philadelphia Times. — The third year of this standard journal inaugurates a change in its issue which will be received with pleasure by its subscribers. It ceases to be a bi-monthly, and becomes a weekly.

By this change they will be enabled to give their readers a constantly fresh *resume* of the current news of the profession. To meet the increased expense involved, the price is raised to $5.00. Saturday is the weekly publication day.—*The Clinic.*

Operation for Internal Strangulated Hernia.—Prof. G. Marcacci, of Florence, performed gastrotomy in October last, on a young man for the relief of a hernia caused by excessive vomiting. The protrusion occurred in the right iliac region, about three inches above the crural arch, and eight or ten inches from the pubes. Ten days following the operation the patient was able to walk, and was discharged cured at the end of a month.—*Lo Sperimentale.—Med. Record.*

Small-Pox in Vienna. — The epidemic continues to spread. The number of patients averages 400; the daily mortality 12, among whom many are adults.— *Wien. Med. Presse.*

The Intra-Venous Injection of Chloral.—The following are the conclusions of a series of articles lately running through the Paris press, with detailed experiments, etc., on the action of chloral by intra-venous injection: By M. Ore, Surgeon to the hospital St. Andre.

1. The injection of chloral into the veins is attended by effects much more rapid and always more durable than is obtained by introduction of this agent into the digestive passages.

2. Chloral injected into the veins in combat of the tetanic phenomenon caused by strychnia, neutralizes the action of this alkaloid to that degree as to render it nil.

3. I am justified, then, in considering chloral as the antidote to strychnia.

4. As the last experiments mentioned prove that chloral injected into the veins checks the so rapidly fatal effects of strychnia when intraduced directly into the veins or administered hypodermically, it is probable that chloral is a remedy of curative efficacy in the treatment of tetanus.

5. I do not hesitate to think that the employment of this agent, according to our method, will give the same results in the treatment of convulsive affections, even of hydrophobia. I have already commenced, to settle this point, some experiments which I will communicate to the society at a later date.

6. This method of medicated injections into the veins is absolutely innocuous.— *Gaz. des Hop.*, Sept. 7, '72.

An Ointment for Neuralgia.—Dr. J. Knox Hodge recommends the following as an application which will relieve facial or any other neuralgia almost instantaneously. He advises his friends to "send around to the druggist's and try a bottle."

℞—Albumen of egg, ʒ j.
Rhigolene, ℥ iv.
Oil of peppermint, ℥ ij.
Collodion,
Chloroform, aa ʒ j.—M.

Agitate occasionally for 24 hours, and by gelatinization a beautiful semi-solidified opodeldoc-looking compound results, which will retain its consistency and hold the ingredients intimately blended for months.

In the above we find a local agent of signal potency in all neuralgic affections, and for the relief of pain generally.

Apply by smart friction with the hand, or gently with a soft brush or mop along the course of the nerve involved.— *Georgia Companion*, Aug., '72.

Excision of the Left Scapula. — Samuel Logan, M. D., New Orleans, La. (*Richmond and Louisville Med. Journal*, August, 1872), publishes a case of excision of the left scapula subsequently to resection of the head of the humerus of the same side. He is agreeably surprised at the degree of usefulness of the extremity after such an extensive removal of bone. The patient, male, aged 33 years, during convalescence from small-pox received severe blows on his shoulders, and shortly after began to suffer pain in the left scapulo-humeral joint, followed by caries of the head of the humerus and body of the scapula.

Three months after the operation the wound had almost entirely healed, with the following use of the limb: He could lift readily any moderately heavy article; could pull perpendicularly (lifting) 26 pounds, horizontally, 26½ pounds; by simply flexing the forearm (and not lifting with the body) he could raise 14 pounds; could place his hand on either ear or shoulder, but not on back of the neck, and pass his hand over the whole of his forehead and face; he could not carry his hand behind his back, but could readily bring it so as to sweep over the whole chest and face. The cavity left by the operation was filled almost to the level of the surrounding parts with a somewhat hard mass of new deposit.

A Floating Hospital for Chest Diseases.—To our numerous land hospitals low down on the sea coast and high up in the mountains, is to be added now a new institution with very many advantages, but with the single disadvantage of being only for the benefit of the rich. Dr. V. Wallendorf establishes a hospital for chest diseases this winter on a screw propeller, which is to journey out late in the fall from Cuxhaven beyond Gibraltar and along the coast to Malta, if the weather is fair, and to return again in the beginning of April. The vessel is to be specially prepared for its purpose, is to have a competent physician on board, and the patients are to be allowed to make land sorties as long as possible in several stations of mild climate.— *Wien. Med. Presse*, Sept. 1, '72.

Singular Case of Hemorrhage from the Bowels. — A rare and important case is reported in the *Bulletin de Therapeutique*, June 30, which throws light on some hitherto inexplicable cases of sudden death from intestinal hemorrhage. A man, 74 years of age, had enjoyed good health, except a tendency to constipation. One night he was taken with intestinal hemorrhage, and died in a few hours. Only twice, and that about a month before, had he had slight attacks of a similar character.

A careful autopsy disclosed the only lesion to be *erectile tumor*, the size of an almond, in the mucous coat of the duodenum. It was composed merely of an enlargement of the capillary vessels of the part, or so-called angioma. A slight ulceration at its edge had given rise to the hemorrhage which destroyed life.—*Med. and Surg. Reporter.*

Ingrowing Toe-Nail. — Dr. Finch writes to the *British Medical Journal*:—

Neither of the cutting operations is at all necessary for the complete and rapid cure of ingrowing toe-nail. If a small, thin, flat piece of silver plate be bent at one edge into a slight, deep groove, and, after the toe has been poulticed twenty-four hours, slipped beneath the edge of the nail, so as to protect the flesh from its pressure, and the rest of the plate bent round the side and front of the toe, being kept in position with a small portion of resin plaster passed round the toe, a speedy and almost painless cure will take place; and the patient, after the first day, has the additional advantage of being able to walk. I have followed this method in numerous cases with uniform success.—*Ibid.*

Vaccination a Proof Against Small-Pox.—James F. Weeds, Surgeon U. S. Army, in a valuable paper on "Vaccination," etc., firmly believes, with a majority of the profession, that vaccination and re-vaccination properly performed, renders complete protection against small-pox, and that wherever the disease exists, it is in consequence of this operation having been insufficiently and improperly performed. Epidemics of small-pox find their victims among the unvaccinated, and the imperfectly and insufficiently vaccinated. Wipe out these classes, and at the same time epidemic small-pox will be wiped out. The fault is not in the defective power of vaccination, but in the imperfect and incomplete manner in which it is used. —*The Medical Record.*

Muscle as a Constituent of Nerve-Tissue. — Tigri has shown the presence of muscular fibers among the nerve-fibers of the cerebro-spinal nerves, and the ganglia of the sympathetic. When an irritation is applied, contraction is caused, and the liquid contents of the nerve-fibers are caused to vibrate, and the motion is conveyed thus centripetally or centrifugally. In reference to these anatomical and physiological peculiarities, he calls the ganglia of the sympatheticus magnus, as well as the large ganglia of the brain, "nerve hearts." He considers that the so-called Remak's nerve-fibers are muscular fibers. —*N. Y. Med. Jour.*

Blisters in Pneumonia. — Dr. C. J. B. Williams, in speaking of pneumonia, says: "My experience has taught me to put great faith in large blisters, both in asthenic pneumonia and bronchitis, and I am confident that I have seen many lives saved by their means. Instead of being lowering, they give a salutary excitement to the circulation, and the copious serous discharge which proceeds from the skin tends to relieve the congested lung without wasting the red blood, that is so necessary to sustain the functions. Small blisters tease as much as large ones, and are far inferior in the relief they afford.—*American Practitioner*.

Effect of Ether on Uterine Contractions.—At a late meeting of the Obstetrical Society of Boston, Dr. Abbott (*Boston Med. and Surg. Journal*, August 15, 1872,) mentioned a case of labor, in which uterine contractions ceased after the full use of ether; although it has been said that anæsthetics in labor never interfere with the expulsive action of the uterus.—*Ibid.*

